# Evaluation of *p53* and Its Target Gene Expression as Potential Biomarkers of Cholangiocarcinoma in Thai Patients

**DOI:** 10.31557/APJCP.2020.21.3.791

**Published:** 2020-03

**Authors:** Janpen Puetkasichonpasutha, Nisana Namwat, Prakasit Sa-Ngiamwibool, Attapol Titapun, Tuangporn Suthiphongchai

**Affiliations:** 1 *Department of Biochemistry, Faculty of Science, Mahidol University, Bangkok, *; 2 *Department of Biochemistry, *; 4 *Department of Pathology,*; 5 *Department of Surgery, Faculty of Medicine, *; 3 *Cholangiocarcinoma Research Institute, Khon Kaen University, Khon Kaen, Thailand. *

**Keywords:** Cholangiocarcinoma- Clinicopathology- Plasminogen activator inhibitor-1- tumor suppressor *p53*- WIP1

## Abstract

**Background:**

Cholangiocarcinoma (CCA), a common cancer in northeastern Thailand, is a severe disease with poor prognosis and short survival time following diagnosis. DNA damage in CCA is believed to be caused by liver fluke infection in combination with exposure to carcinogens. *p53*, a tumor suppressor, is the most mutated gene in human cancers including liver fluke-associated CCA. Hence, expression patterns of *p53* and its target genes may be useful for diagnosis and/or prognosis of CCA patients.

**Methods:**

Differential mRNA expression of *p53* and its target genes, namely, *FUCA1*, *ICAM2 MDM2*, *p21*, *PAI-1*, *S100A9*, and* WIP1* in CCA tissue samples (n = 30) relative to matched adjacent non-tumor tissues was determined by quantitative RT-PCR and compared to clinicopathological features. Level of p53 protein was determined by immunohistochemistry and correlated with the expression of its target genes.

**Results:**

Immunohistochemistry showed elevation of p53 protein level in 77% of the cases, while RT-PCR showed downregulation of p53 mRNA and its seven target genes in 23% and 47-97% of the samples. *PAI-1* was down-regulated in almost all CCA samples, thus highlighting it as a potential diagnostic marker for CCA. However, no significant clinical associations were found except for down-regulation of WIP1 that was significantly correlated with non-papillary type tissue (p-value = 0.001) and with high p53 protein level (p-value = 0.007).

**Conclusion:**

Our results demonstrated statistically significant association between down-regulation of WIP1 with non-papillary type and with high p53 protein level, and PAI-1 was down-regulated in almost all CCA. Therefore, expression level of *WIP1* and *PAI-1* may be useful for predicting p53 functional status and as a potential diagnostic marker of CCA, respectively.

## Introduction

Cholangiocarcinoma (CCA) is a malignant tumor arising from cells within the bile duct (Hammill and Wong, 2008). As CCA is asymptomatic at the early stages, detection of patients with this cancer usually occur at the metastatic stage, when tumor removal has minimal effect on survival outcome, a median ranging from 12 to 40 months and five-year survival rate of 14 to 36% respectively (Guglielmi et al., 2009; Woradet et al., 2016). To date, there is no specific biomarker for early detection of CCA (Bartella and Dufour, 2015; Loosen et al., 2018). Although this type of cancer is rare worldwide, it is notably prevalent in northeastern Thailand, which also is an endemic of liver fluke *Opisthorchis viverrini *(OV) infection, one of the suspected etiologies of CCA (Sripa et al., 2011). Infection with OV through consuming raw freshwater fish infected with the parasite cysts ultimately results in infection with adult forms of OV at the bile duct, where the presence of OV secretory carcinogenic products, mechanical damage and chronic inflammation are thought to induce pathological changes to host cells, such as DNA damage, interference to DNA repair mechanisms and/or apoptosis, ultimately resulting in genetic alterations and malignant transformation of cholangiocytes (Sripa et al., 2012). 

Tumor suppressor *p53*, “guardian of the genome”, is a sequence-specific transcription factor (Funk et al., 1992; Lane, 1992; Pietenpol et al., 1994) playing a key role in prevention of tumor development by regulating expression of its downstream target genes involved in cell cycle arrest, DNA repair, apoptosis and cell senescence (Vousden and Lu, 2002; Kastenhuber and Lowe, 2017). Loss-of-function mutations in* p53* are found in more than 50% of human cancers (Kandoth et al., 2013) and is also the most prevalent mutated gene in OV-associated CCA (Ong et al., 2012). In addition to loss of native function, heterozygous mutant* p53 *can also have dominant negative effects over wild-type protein function, thereby compromising expression of its downstream target genes (Billant et al., 2016; Vieler and Sanyal, 2018). Among the gene targets of *p53*, the most predominant is that encoding cyclin-dependent kinase (CDK) inhibitor p21, a negative regulator of cell cycle progression (Karimian et al., 2016). Another common p53 target is MDM2, an E3 ubiquitin-protein ligase, which negatively regulates p53 function through a proteasome degradation process (Urso et al., 2016). During DNA damage, p53 and MDM2 are phosphorylated, resulting in a decrease in p53-MDM2 interaction, thereby reducing p53 degradation, and together with a concomitant increase in p53 production result in a net increase in p53 (Cheng et al., 2009).

Wild-type p53-inducible phosphatase 1 (WIP1), of which expression is stimulated in response to γ or UV radiation in a p53-dependent manner (Fiscella et al., 1997), suppresses transcriptional and apoptotic activities of p53 through reduction of p38 MAP kinase-mediated p53 phosphorylation and promotes MDM2-mediated p53 degradation (Takekawa et al., 2000; Lu et al., 2007). In addition p53 also mediates metastasis through regulating expression of PAI (Shetty et al., 2008), an inhibitor of tPA and uPA, key secreted proteases in cancer metastasis (Binder et al., 2002). 

Other than these well-known *p53* target genes, there exists a number of novel p53-target genes associated with tumorigenesis, viz. α-L-fucosidase-1 (FUCA1), encoded a lysosomal protein involved in cancer progression by suppressing cancer cell growth and inducing cell death (Ezawa et al., 2016); S100 calcium-binding protein A9 (S100A9), an inflammatory marker inducing apoptosis through mediating p53-dependent apoptotic pathway (Li et al., 2009), with down-regulation of S100A9 associated with squamous cell carcinoma of head and neck (Khammanivong et al., 2016), esophagus (Kong et al., 2004; Pawar et al., 2015) and nasopharynx (Fung et al., 2000); and intercellular adhesion molecule-2 (ICAM2), a type I transmembrane protein activated by p53 to function as a tumor suppressor by inducing immune response through accumulation of immature myeloid dendritic cells in pancreatic carcinogenesis (Hiraoka et al., 2011), and by down-regulation of ICAM2 associating with cancer progression and metastasis (Sasaki et al., 2016).

Although direct DNA sequencing or mutation assays are the gold standard for detecting *p53* mutation, it is laborious and time consuming. On the contrary, immunohistochemistry (IHC) is an economic and convenient method. Additionally, overexpression of p53 protein detected by IHC have been proven to be useful for predicting *p53* mutation in hepatocellular carcinoma (Liu et al., 2016). Besides, p53 alterations have an effect on its downstream targets. Hence, the expression of these p53 target genes might indicate the status of p53 in cancer and their contributions to various steps of cancer progression. In this study, mRNA expression levels of p53 and the seven above-mentioned p53 target genes in CCA and adjacent non-tumor (NT) tissues were investigated and correlated with clinicopathology. Furthermore, p53 protein was determined by IHC and correlated with p53 target gene expression. Any significant association of these genes with clinicopathology data may help identify possible diagnostic or prognostic biomarkers of CCA tumorigenesis.

## Materials and Methods

*Human CCA and Adjacent Non-Tumor (NT) Tissues*

Sixty frozen liver tissues (30 CCA and 30 NT) from CCA patients who had liver resection were provided by Cholangiocarcinoma Research Institute, Faculty of Medicine, Khon Kaen University, Khon Kaen, Thailand. Average age of the patients is 58 ± 10 years with the average survival time of 1.4 ± 1.2 years. Histological properties of the tissues were examined by a certified pathologist with histological grading classified as well-, moderate-differentiated stage (Nakanuma et al., 2000).

The study protocol was approved by Ethics Committee of Khon Kaen University (ethical clearance no. HE571283).

*Quantitative (q) RT-PCR*

RNA was extracted from liver tissue using TRIzol^TM^ Reagent (Invitrogen, Carlsbad, CA, USA). In brief, liver tissues were homogenized in TRIzol^TM^ Reagent and RNA was extracted with phenol/chloroform solution, precipitated with isopropanol and dissolved in RNase-free water. RNA concentration was determined with a Nano Drop spectrophotometer (Thermo Fisher Scientific, Waltham, MA, USA). QRT-PCR was performed by incubating 2 μg of RNA with 0.5 μg of random oligohexamer, 0.5 mM dNTP and 160 U ImProm-II™ reverse transcriptase (Promega, South-Central, WI, USA) in a 20-μl reaction containing 3 mM MgCl_2_ and 1X reaction buffer (Promega) for 60 min at 42°C. Then qPCR amplification was performed in a 10-μl mixture containing 1X FastStart Universal SYBR Green Master Mix (Roche Diagnostics, Rotkreuz, Switzerland), 25 ng of cDNA, 2 pmol of each specific primer pair (Table 1) in a CFX Connect™ Real-Time PCR Detection System (Bio-Rad, Richmond, CA, USA) under the following thermocycling conditions: 95°C for 10 min, followed by 40 cycles of 95°C for 15 sec and 55°C for 1 min. Adjusted Ct values with rounding up was used in the calculating expression level of target gene in CCA relative to matched NT tissues samples using 2^-^^∆∆^^Ct^ formula (normalized with glyceraldehyde 3-phosphate dehydrogenase gene expression).

*Immunohistochemistry*

Paraffin-embedded human liver CCA and matched NT tissues samples were deparaffinized by autoclaving for 10 min and then rehydrated with 10 mM sodium citrate buffer containing 0.05% Tween and 0.05% triton X. Tissue endogenous peroxidase activity was inhibited by treating with 0.3% (v/v) hydrogen peroxide for 30 min. Following incubation with 10% skim milk for 30 min, tissue sections were probed with primary antibody, murine primary anti-p53 antibodies (sc-126; Santa Cruz Biotechnology, Dallas, TX, USA) followed by peroxidase-conjugated Envision™ secondary anti-mouse antibody (DAKO, Glostrup, Denmark). Visualization of immunoreactive p53 was performed using a 3,3′-diaminobenzidine tetrahydrochloride (DAB) substrate kit (Vector Laboratories, Inc., CA, USA), followed by counter staining with Mayer’s hematoxylin. Tissue sections were dehydrated with an increasing concentration of ethanol solution and mounted with permount solution. Negative control slides were similarly prepared but without primary antibody treatment. IHC score was calculated by multiplying frequency of positive staining (0 = <10%: 1 = 10-25%, 2 = 25-50%, and 3 = 50-100%) with staining intensity score (0 = negative, 1 = weak, 2 = moderate, and 3 = strong staining). Samples with IHC score either in nucleus or in cytoplasm higher than median were classified as high p53, and those with IHC score both in nucleus and cytoplasm lower than median were low p53.

*Statistical Analysis*

Correlation between relative expression of* p53*-target genes with clinicopathological features and among p53 levels in CCA tissue with gene expression and clinicopathological parameters were analyzed using Fisher’s Exact probability test. Patient survival analysis was evaluated using Kaplan–Meier method and compared employing a log-rank test. Statistical analysis was carried out using SPSS version 22.0 (SPSS Inc., Chicago, IL, USA) and result is considered statistically significant when *P*-value <0.05.

## Results

*Expression of p53 and p53 target genes in human CCA tissues and NT*

**Figure 1:**
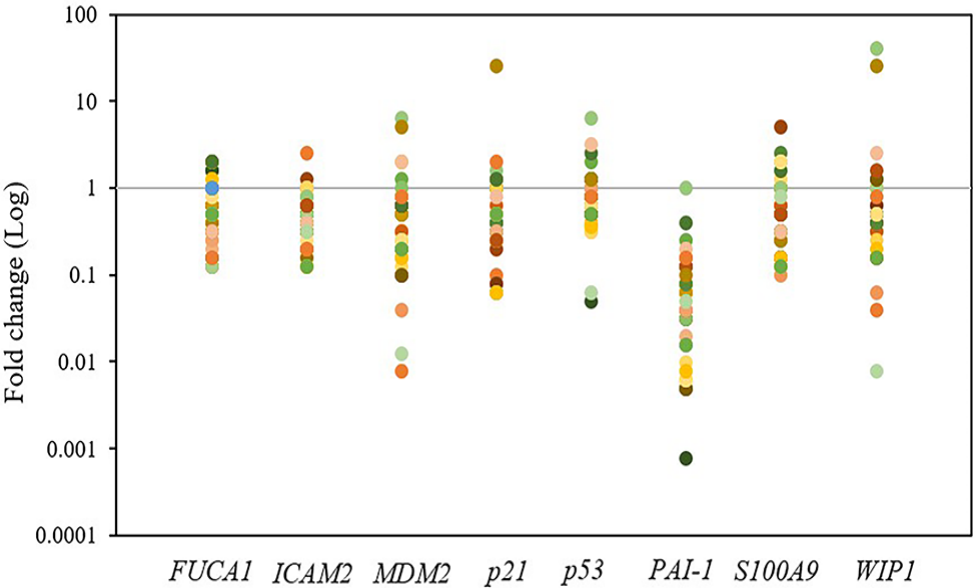
Gene Expression in Cholangiocarcinoma (CCA) Compared to Matched Adjacent Non-tumor (NT) Tissues. Quantitative RT-PCR was employed to determine mRNA level of in CCA relative to NT tissue samples (n = 30) using 2^-^^δδ^^Ct^ formula (normalized with glyceraldehyde 3-phosphate dehydrogenase gene expression). Each colored dot represents the same sample. Ct, threshold cycle

**Figure 2:**
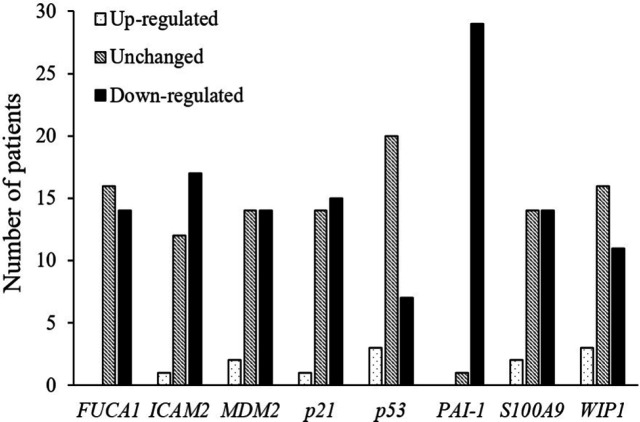
Comparison of Gene Expression in Cholangiocarcinoma Samples Compared to Matched Adjacent Non-tumor (NT) Tissues (n = 30). The bar graph shows number of patients with up-regulated (fold difference >2), down-regulated (fold difference <0.5) or Unchanged (fold difference in the range of 0.5-2.0) in mRNA expression in CCA tissues compared to NT

QRT-PCR was performed to determine mRNA expression levels of* p53* and its seven target genes, namely, *FUCA1*,* ICAM2*, *MDM2*, *p21*, *PAI-1*, *S100A9*, and *WIP1*, in human CCA tissues (n = 30) relative to matched NT tissues. Relative up-regulation (>2-fold difference) in mRNA expression levels between CCA tissues and NT of p53 or its target genes was found only in a few cases (0-3 cases (0-10%); 2.5-40.3 folds), while the down-regulation (< 0.5-fold difference) was in majority of the cases (up to 29 cases (97%); 0.00-0.40 fold) (Figure 1). PAI-1 gene expression was down-regulated the most (97% of CCA tissues) (Figure 2), while expression of *FUCA1*, I*CAM2*, *MDM2*, *p21*, *p53*, *S100A9*, and *WIP1* genes was down-regulated in 14 (47%; 0.13- to 0.40-fold), 17 (57%; 0.13- to 0.4-fold), 14 (47%; 0.01- to 0.31-fold), 15 (50%; 0.06- to 0.40-fold), 7 (23%; 0.05- to 0.40-fold), 14 (47%; 0.10- to 0.31-fold), and 11 (37%; 0.01- to 0.40-fold) cases, respectively (Figure 1 and Figure 2).

*Correlation between p53 target gene expression and clinicopathological features and survival of CCA Patients*

**Figure 3:**
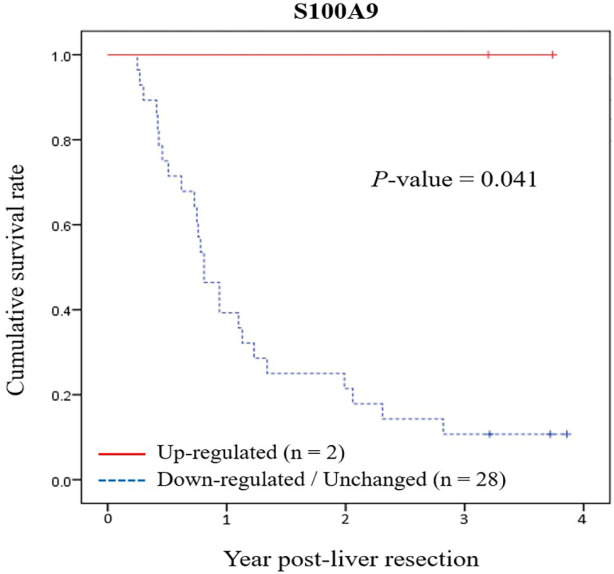
Kaplan-Meier Survival Plot of Association of Cholangiocarcinoma S100A9 Expression and Patient’s Survival Post-liver Resection

Analysis of the associations of mRNA expression levels of* p53* and its seven target genes with clinicopathological features revealed down-regulation of *WIP1* gene expression is significantly correlated with non-papillary type (*P*-value = 0.001), while there is no correlation between *WIP1* gene expression and other clinicopathological features (Table 2). Although *S100A9* up-regulated gene expression is significantly correlated with better survival (*P*-value = 0.005) (Table 2) and Kaplan-Meier plot demonstrated a significant correlation between *S100A9* up-regulated gene expression and longer patient’s survival (*P*-value = 0.041) (Figure 3), it is still too early to make any conclusion due to the limited number of samples. In addition, no correlation between *S100A9* up-regulated gene expression and other clinicopathological features could be discerned. Although more than 23% of CCA patients exhibited down-regulation of *FUCA1*, *ICAM2*, *MDM2*, *p21*, *p53*, or *PAI-1* expression, there is no correlation between expression of these genes and various clinicopathological parameters.

*Expression of p53 protein and its correlation with the mRNA expression of p53 and its downstream target genes*

**Figure 4:**
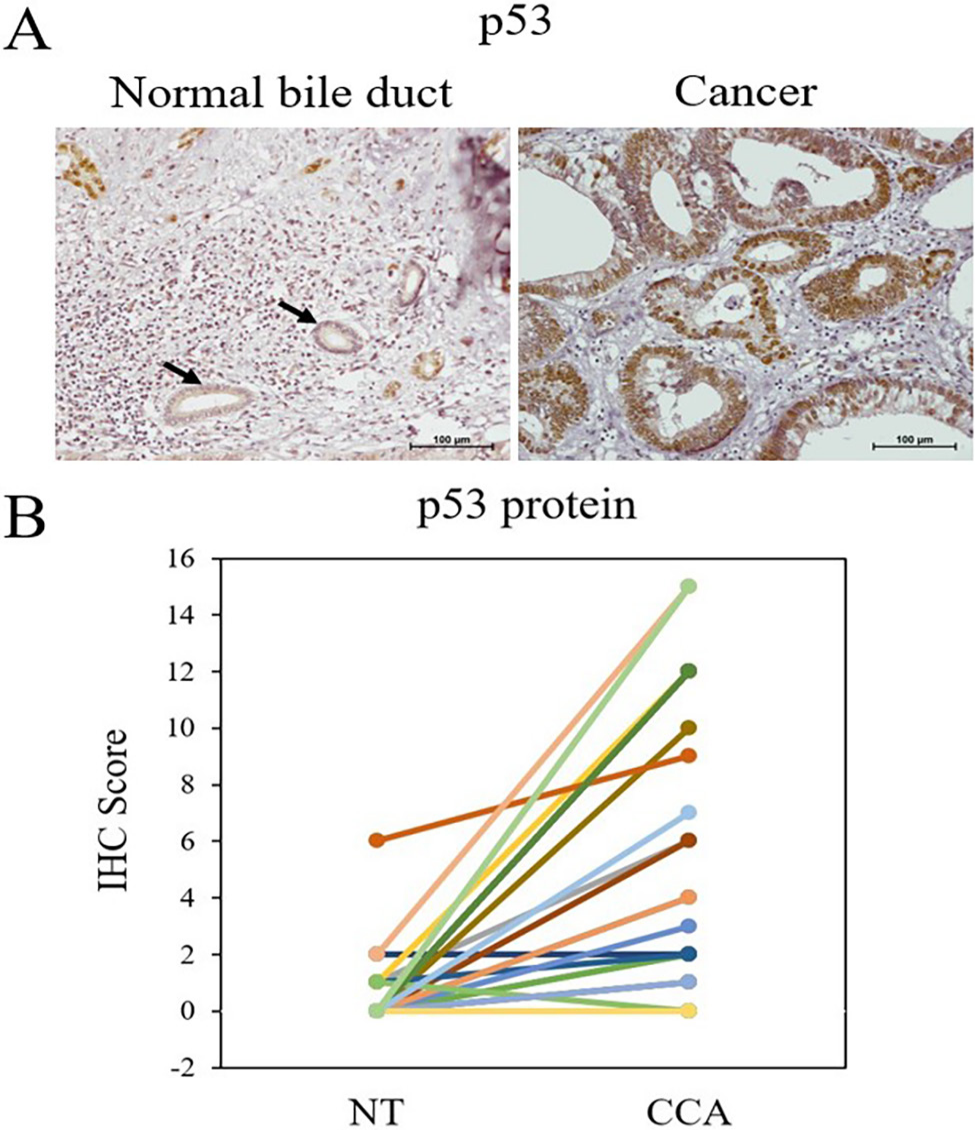
p53 Immunohistochemical Staining in Normal Bile Duct of an Adjacent Non-tumor Area and CCA Tissues. (A) Representative of IHC staining (magnification, x200). Arrows are indicated normal bile duct. (B) The distribution of p53 IHC scores calculated by combining IHC scores in nucleus and in cytoplasm in human CCA tissues and matched NT tissues (n = 30).

Wild-type *p53* generally has a short half-life making it unable to be detected within the cell (Vijayakumaran et al., 2015). However, mutant *p53*, often found in human CCA, accumulates in tumor cells and is able to be detected by such technique as IHC. Thus, p53 protein level could reflect the mutational status of *p53*. In this study, IHC revealed overexpression of p53 in 23 (77%) cancer tissues when compared with NT (Figure 4A). The IHC scores of p53 protein in cancer regions (6 ± 1) were mostly higher than those in normal (0.5 ± 0.2) (Figure 4B). As p53 function in regulation of its target gene expression, levels of its target gene expression should be able to imply p53 functional status. Studying the association of p53 protein and its downstream target mRNA expression demonstrated that high p53 protein level was significantly correlated with down-regulated *WIP1* expression (*P*-value = 0.007) (Table 3), but no association of p53 level was discernable with any clinicopathological features.

**Table 1:** Primers Used in the Study

Primer set	Direction	Sequence (5' → 3')	Amplicon (bp)	Reference
FUCA1	Forward	AGTCACCCTGTTGCCTATGG	190	[44]
	Reverse	TTTGGCGCTTTTAGATTGCT		
GAPDH	Forward	CACCAGGGCTGCTTTTAACTCTGGTA	131	[45]
	Reverse	CCTTGACGGTGCCATGGAATTTGC		
ICAM2	Forward	AGGTACACGTGAGGCCAAAG	179	
	Reverse	CGTGTCATGGGAGATGTTTG		
MDM2	Forward	GCAGTGAATCTACAGGGACGC	83	[46]
	Reverse	ATCCTGATCCAACCAATCACC		
p21	Forward	GCAGACCAGCATGACAGATTT	70	[47]
	Reverse	GGATTAGGGCTTCCTCTTGGA		
p53	Forward	CCCCTCTGAGTCAGGAAACA	151	
	Reverse	TCATCTGGACCTGGGTCTTC		
PAI-1	Forward	GGCCATTACTACGACATCCTG	150	
	Reverse	GGTCATGTTGCCTTTCCAGT		
S100A9	Forward	TGGAGGACCTGGACACAAATG	109	[48]
	Reverse	TCGTCACCCTCGTGCATCTT		
WIP1	Forward	ATCCGCAAAGGCTTTCTCGCTT	61	[49]
	Reverse	TTGGCCATTCCGCCAGTTTCTT		

**Table 2:** Clinicopathological Features of Cholangiocarcinoma (CCA) Patients (n = 30) and Expression of p53 and Its Seven Target Genes in CCA (Relative to Matched Adjacent Normal) Tissue Samples

Clinicopathological parameters	*FUCA1*					*ICAM2*				*MDM2*				*p21*			
	n	Down- regulated	Unchanged	Up- regulated	*P-*value*	Down- regulated	Unchanged	Up- regulated	P-value*	Down- regulated	Unchanged	Up- regulated	*P-*value*	Down- regulated	Unchanged	Up- regulated	*P-*value*
		14 (46.7%)	16 (53.3%)	0 (0.0%)		17 (56.7%)	12 (40.0%)	1 (3.3%)		14 (46.7%)	14 (46.7%)	2 (6.7%)		15 (50%)	14 (46.7%)	1 (3.3%)	
Survival (n=30)																	
Survived <3 years	25	13	12	0	0.336	14	10	1	1.000	12	11	2	1.000	11	13	1	0.441
Survived ≥3 years	5	1	4	0		3	2	0		2	3	0		4	1	0	
Histology type (n=30)																	
papillary type	17	8	9	0	1.000	8	9	0	0.182	5	11	1	0.056	7	10	0	0.193
Non-Papillary type	13	6	7	0		9	3	1		9	3	1		8	4	1	
Histology grading (n=13)																	
Well differentiation	12	6	6	0	1.000	8	3	1	0.385	7	4	1	1.000	8	3	1	1.000
Moderate differentiation	1	1	0	0		0	1	0		1	0	0		1	0	0	
Metastasis (n=30)																	
No metastasis	18	7	11	0	0.457	11	6	1	0.683	10	7	1	0.618	8	10	0	0.338
Metastasis	12	7	5	0		6	6	0		4	7	1		7	4	1	
		7 (23.3%)	20 (66.7%)	3 (10.0%)		29 (96.7%)	1 (3.3%)	0 (0.0%)		14 (46.7%)	14 (46.7%)	2 (6.7%)		11 (36.7%)	16 (53.3%)	3 (10.0%)	
Survival (n=30)																	
Survived <3 years	25	7	15	3	0.426	24	1	0	1.000	11	14	0	0.005*	9	13	3	1.000
Survived ≥3 years	5	0	5	0		5	0	0		3	0	2		2	3	0	
Histology type (n=30)																	
papillary type	17	2	13	2	0.275	16	1	0	1.000	7	10	0	0.147	2	14	1	0.001*
Non-Papillary type	13	5	7	1		13	0	0		7	4	2		9	2	2	
Histology grading (n=13)																	
Well differentiation	12	4	7	1	0.462	12	0	0	-	6	4	2		7	3	2	1.000
Moderate differentiation	1	1	0	0		1	0	0		1	0	0	1.000	1	0	0	
Metastasis (n=30)																	
No metastasis	18	4	11	3	0.445	17	1	0	1.000	9	8	1		6	10	2	0.869
Metastasis	12	3	9	0		12	0	0		5	6	1	1.000	5	6	1	

*The comparison of variables that had 3 categories, significance at ≤ 0.05 using Fisher’s exact probability test

**Table 3:** Correlation between p53 Protein Expression (by IHC) and mRNA Expression of p53 and Its Seven Target Genes in CCA Tissue Samples

mRNA expression		p53 protein^#^			mRNA		p53 protein^#^		P-value*
	n	Low expression 9 (30%)	High expression 21 (70%)	P-value*	expression	n	Low expression 9 (30%)	High expression 21 (70%)	
	(n = 30)					(n = 30)			
*p53*					*PAI-1*				
Down-regulated	7	1	6	0.829	Down-regulated	29	9	20	1.000
Unchanged	20	7	13		Unchanged	1	0	1	
Up-regulated	3	1	2		Up-regulated	0	0	0	
*p21*					*FUCA1*				
Down-regulated	15	3	12	0.204	Down-regulated	14	6	8	0.236
Unchanged	14	5	9		Unchanged	16	3	13	
Up-regulated	1	1	0		Up-regulated	0	0	0	
*MDM2*					*S100A9*				
Down-regulated	14	3	11	0.580	Down-regulated	14	6	8	0.376
Unchanged	14	5	9		Unchanged	14	3	11	
Up-regulated	2	1	1		Up-regulated	2	0	2	
*WIP1*					*ICAM2*				
Down-regulated	11	0	11	0.007*	Down-regulated	17	4	13	0.596
Unchanged	16	7	9		Unchanged	12	5	7	
Up-regulated	3	2	1		Up-regulated	1	0	1	

^#^p53 protein with IHC score either in nucleus or in cytoplasm higher than median were classified as high p53, and those with IHC score both in nucleus and cytoplasm lower than median were low p53; ^*^The comparison of variables that had 3 categories, significance at ≤ 0.05 using Fisher’s exact probability test.

## Discussion

CCA is usually detected at an advanced stage leading to poor prognosis. The major risk factors of CCA include OV infection, nitrosamines and hepatitis C virus infection (Sripa et al., 2007; Li et al., 2015), a combination of which was suggested to induce DNA damage in bile duct cells (Sripa et al., 2007). In particular, loss-of-function mutations in p53 would result in uncontrolled cell proliferation, resistance to apoptosis and accumulation of genetic alterations (Vijayakumaran et al., 2015). Wild type p53 has a short half-life, thus difficult to detect by IHC, while mutant forms are usually more stable and can readily be detected, e.g. IHC (Vijayakumaran et al., 2015). As p53 exerts its biological activities through transcriptional regulation of its target genes, mutations affecting* p53* function could be implied from changes in expression levels of these target genes.

In the present study cellular *p53* content determined by IHC was overexpressed in 77% of CCA tissue samples compared to match NT tissue, suggestive of carriage of (putative) mutant *p53*. Furthermore, 37-97% of mRNA levels of seven *p53* target genes were down-regulated in CCA tissues compared to those in matched adjacent NT specimens, while only 23% of p53 mRNA was down-regulated. Thus, besides down-regulation of p53 expression, the discrepancy of *p53* target genes might result from functional defective mutant *p53* overexpressed in CCA.

However, there was a notable lack of significant associations between decreased *p53* mRNA expression, elevated (putative mutant) p53 content or down-regulated expression of *p53* target genes with clinicopathological features, except in the case of *WIP1*, decreased expression of which was associated with non-papillary type. Although our data also showed statistically significant increased survival rate of CCA patients in association with up-regulation of S100A9, the number of up-regulated cases were too low to make such conclusion. Regarding *WIP1*, a target of *p53* and also a *p53* negative regulator, its over-expression was reported in many cancer types, and leading to colorectal cancer progression (Li et al., 2013) and kidney cancer metastasis (Sun et al., 2015). In cancer cells with wild-type p53, knockdown of *WIP1 *expression enhances doxorubicin-induced apoptosis via Bax-dependent p53 activation (Kong et al., 2009). In CCA, down-regulation of WIP1 is associated with non-papillary type, generally a poor prognosis in intrahepatic CCA (Jarnagin et al., 2005). However, these adverse clinical outcomes were not evident in our study.

The above mention associations of *S100A9* and *WIP1 *expressions with clinicopathological features suggest their potential biomarkers of CCA progression, but the number of cases are too limited to make any conclusion. It is of note that PAI-1 was down-regulated in 97% of CCA tissue samples, thus highlighting its loss in the etiology of CCA and its potential diagnostic marker of CCA.

In conclusion, we showed that p53 protein is elevated in most CCA tissue samples and inversely correlated with *WIP1 mRNA* expression. Down-regulation of *WIP1 *associated with non-papillary type, and down-expression of *PAI-1* was present in almost all CCA tissue samples. Although this study was limited by the small sample number, it provides some trends of potential biomarkers of CCA.
